# Phagocytosis is a primary determinant of pulmonary clearance of clinical *Klebsiella pneumoniae* isolates

**DOI:** 10.3389/fcimb.2023.1150658

**Published:** 2023-03-28

**Authors:** Rick van der Geest, Hongye Fan, Hernán F. Peñaloza, William G. Bain, Zeyu Xiong, Naina Kohli, Emily Larson, Mara L. G. Sullivan, Jonathan M. Franks, Donna B. Stolz, Ryota Ito, Kong Chen, Yohei Doi, Melanie J. Harriff, Janet S. Lee

**Affiliations:** ^1^ Acute Lung Injury Center of Excellence, Division of Pulmonary, Allergy, and Critical Care Medicine, Department of Medicine, University of Pittsburgh, Pittsburgh, PA, United States; ^2^ Veterans Affairs (VA) Pittsburgh Health Care System, Pittsburgh, PA, United States; ^3^ Veterans Affairs (VA) Portland Health Care System, Portland, OR, United States; ^4^ Department of Cell Biology, Center for Biologic Imaging, University of Pittsburgh, Pittsburgh, PA, United States; ^5^ Department of Respiratory Medicine, Japanese Red Cross Aichi Medical Center Nagoya Daiichi Hospital, Nagoya, Japan; ^6^ Division of Infectious Diseases, Department of Medicine, University of Pittsburgh, Pittsburgh, PA, United States; ^7^ Departments of Microbiology and Infectious Diseases, Fujita Health University, Toyoake, Japan; ^8^ Division of Pulmonary, Allergy and Critical Care Medicine, Department of Medicine, Oregon Health State University, Portland, OR, United States; ^9^ Vascular Medicine Institute, University of Pittsburgh, Pittsburgh, PA, United States; ^10^ Division of Pulmonary and Critical Care Medicine, Washington University in St. Louis, St. Louis, MO, United States

**Keywords:** respiratory infection, host defense, Klebsiella pneumoniae, phagocytosis, clinical isolates, macrophages

## Abstract

**Introduction:**

*Klebsiella pneumoniae* (*Kp*) is a common cause of hospital-acquired pneumonia. Although previous studies have suggested that evasion of phagocytic uptake is a virulence determinant of *Kp*, few studies have examined phagocytosis sensitivity in clinical *Kp* isolates.

**Methods:**

We screened 19 clinical respiratory *Kp* isolates that were previously assessed for mucoviscosity for their sensitivity to macrophage phagocytic uptake, and evaluated phagocytosis as a functional correlate of *in vivo Kp* pathogenicity.

**Results:**

The respiratory *Kp* isolates displayed heterogeneity in the susceptibility to macrophage phagocytic uptake, with 14 out of 19 *Kp* isolates displaying relative phagocytosis-sensitivity compared to the reference *Kp* strain ATCC 43816, and 5 out of 19 *Kp* isolates displaying relative phagocytosis-resistance. Intratracheal infection with the non-mucoviscous phagocytosis-sensitive isolate S17 resulted in a significantly lower bacterial burden compared to infection with the mucoviscous phagocytosis-resistant isolate W42. In addition, infection with S17 was associated with a reduced inflammatory response, including reduced bronchoalveolar lavage fluid (BAL) polymorphonuclear (PMN) cell count, and reduced BAL TNF, IL-1β, and IL-12p40 levels. Importantly, host control of infection with the phagocytosis-sensitive S17 isolate was impaired in alveolar macrophage (AM)-depleted mice, whereas AM-depletion had no significant impact on host defense against infection with the phagocytosis-resistant W42 isolate.

**Conclusion:**

Altogether, these findings show that phagocytosis is a primary determinant of pulmonary clearance of clinical *Kp* isolates.

## Introduction

*Klebsiella pneumoniae* (*Kp*) is a non-motile, Gram-negative bacterium that is commonly found as a commensal in the microbiota of the human gastrointestinal tract and the nasopharynx (Conlan et al., 2012; Gorrie et al., 2017). Classical strains of *Kp* are a common cause of hospital-acquired acute lower respiratory tract infections in immunocompromised or critically ill patients, and these strains are becoming increasingly resistant to antibiotics (Jones, 2010; Munoz-Price et al., 2013; Vincent et al., 2020). In contrast to classical *Kp* strains, hypervirulent *Kp* strains are able to cause infections in otherwise healthy individuals, and over the last few decades these strains have become an important cause of a community-acquired invasive syndrome in Asia (Siu et al., 2012; Russo and Marr, 2019). Hypervirulence is typically determined based on clinical features and/or based on a positive string test for a hypermucoviscous phenotype, but there is no clear consensus definition of hypervirulent *Kp* (Harada and Doi, 2018; Gonzalez-Ferrer et al., 2021). This is underscored by the fact that not all hypervirulent *Kp* strains display a hypermucoviscous phenotype, and not all *Kp* strains that are hypermucoviscous are also hypervirulent (Luo et al., 2014; Togawa et al., 2015; Cubero et al., 2016; Catalán-Nájera et al., 2017). Moreover, while characterization of capsule types by serotyping or sequencing of the *wzi* gene has shown that the K1 and K2 capsular serotypes are prevalent among hypervirulent *Kp* strains, non-K1/K2 strains have also been identified among hypervirulent *Kp* isolates, and conversely, not all K1 and K2 strains display a hypervirulent phenotype (Yeh et al., 2007; Yu et al., 2008; Qu et al., 2015; Lee et al., 2016; Lin et al., 2020). The two distinct *Kp* pathotypes highlight that the ability of *Kp* to cause disease in humans is dependent on both pathogen and host characteristics, but validated molecular and functional correlates of *Kp* pathogenicity that take into account host-pathogen interactions are lacking (Catalán-Nájera et al., 2017; Harada and Doi, 2018; Gonzalez-Ferrer et al., 2021). In this light, there is a significant interest in gaining a better understanding of the cellular and molecular determinants of *Kp* pathogenicity, as well as how these factors affect the overall host immune response and clinical outcome following *Kp* infection.

Evasion of phagocytic uptake is considered a major mechanism by which *Kp* escapes the host immune system, and the polysaccharide capsule is viewed as one of the primary virulence factors that facilitates immune evasion of *Kp* (Domenico et al., 1994; Paczosa and Mecsas, 2016). This notion is supported by several lines of evidence, including the observation that the phagocytosis of capsule-deficient mutants of *Kp in vitro* is more efficient than that of wild-type control bacteria, and that capsule-deficient *Kp* mutants are less virulent *in vivo* (Yoshida et al., 2000; Cortés et al., 2002). Similarly, a *Klebsiella pneumoniae* carbapenemase (KPC)-producing *Kp* strain of capsular serotype K41 was shown to be more susceptible to phagocytosis *in vitro* and less virulent *in vivo* compared to the laboratory reference *Kp* strain ATCC 43816 of the capsular serotype K2, which is known to be associated with hypervirulence (Tzouvelekis et al., 2013). Importantly, alveolar macrophages – the sentinel phagocytes in the lung – have been shown to be critical for effective pulmonary defenses against the ATCC 43816 K2 strain (Broug-Holub et al., 1997). Although these studies have provided evidence that evasion of phagocytosis is an important virulence strategy of *Kp*, there are few data available about the contribution of phagocytic evasion to the pathogenicity of clinical *Kp* isolates. As such, it is for example not known whether susceptibility to phagocytosis is a universal feature of non-K1/K2 clinical isolates, and vice versa, whether K1 and K2 isolates are uniformly resistant to phagocytosis. In addition, it was recently reported that clodronate depletion of alveolar macrophages did not affect the clearance of a carbapenem-resistant *Kp* strain of sequence type 258 from the lungs in a mouse model of acute respiratory infection, which suggests that there may be strain-dependent variation in the importance of alveolar macrophages for host defense against pulmonary *Kp* infection (Ahn et al., 2016).

In the current study, we examined the heterogeneity of phagocytic uptake among clinical *Kp* isolates and evaluated susceptibility to phagocytic uptake as a functional correlate of *in vivo* pathogenicity. For this purpose, we screened 19 clinical respiratory *Kp* isolates for their capacity to be phagocytosed *in vitro*, and we compared the host immune response to a phagocytosis-sensitive and a phagocytosis-resistant *Kp* isolate *in vivo*.

## Materials and methods

### Animals

C57BL/6 mice (6-14 week old) were obtained from the Jackson Laboratory (Bar Harbor, ME) and breeding colonies were established at our facilities. All mice were fed a standard chow diet *ad libitum* and were maintained under specific-pathogen-free conditions in accordance with the guidelines of the Division of Laboratory Animal Resources at the University of Pittsburgh. All mice used for experiments were age- and sex-matched. At the end of experiments, mice were euthanized with an overdose of isoflurane followed by cervical dislocation. All procedures involving animals were performed in compliance with the Guide for the Care and Use of Laboratory Animals from the National Research Council (U.S.) Institute for Laboratory Animal Research, and were approved by the University of Pittsburgh Institutional Animal Care and Use Committee (IACUC protocols #20015967 and #21059260)

### Cell culture

RAW 264.7 cells and THP-1 cells were obtained from American Type Culture Collection (ATCC, Manassas, VA) and maintained, respectively, in DMEM (Lonza, Morristown, NJ) or RPMI 1640 (Lonza) supplemented with 10% heat-inactivated newborn calf serum (Hyclone Newborn Bovine Calf Serum, New Zealand Origin, Cytiva Life Sciences, Marlborough, MA), 100 U/mL penicillin and 100 μg/mL streptomycin (Gibco™, Thermo Fisher Scientific, Waltham, MA). THP-1 cells were differentiated overnight in growth medium supplemented with 1 μM phorbol 12-myristate 13-acetate (PMA, Sigma-Aldrich, St. Louis, MO) prior to use in experiments. Mouse peritoneal cells were obtained by lavaging the peritoneal cavity of C57BL/6 mice three times with 5 mL of ice-cold phosphate-buffered saline (PBS, Lonza) supplemented with 0.6 mM EDTA (UltraPure™, Thermo Fisher Scientific) using a Surflash 14G x 2” IV catheter (Terumo Medical Products, Vaughan, Ontario). Collected peritoneal cells were centrifuged at 300 x *g* for 5 minutes. After this, the cells were resuspended in 1X red blood cell lysis buffer (eBioscience, Thermo Fisher Scientific) to lyse erythrocytes and then centrifuged again at 300 x *g* for 5 minutes. Peritoneal cells were then resuspended in DMEM supplemented with 10% heat-inactivated newborn calf serum, 100 U/mL penicillin and 100 μg/mL streptomycin, after which cells were plated on tissue culture plates and allowed to adhere overnight. The next day, peritoneal cells were washed two times with PBS to remove non-adherent cells, after which remaining peritoneal macrophages were used for downstream experiments. Alveolar macrophages were obtained by lavaging mouse lungs through the trachea five times with 0.8 mL mL of ice-cold PBS supplemented with 0.6 mM EDTA (Thermo Fisher Scientific) using a SurFlash 18G x 1^1^/_4_ IV catheter (Terumo Medical Products) and processed in the same manner as described above for peritoneal lavage cells.

### Bacterial culture

The clinical *Kp* isolates have been previously described (Ito et al., 2015). Bacteria were grown overnight at 37°C in tryptic soy broth (TSB, Sigma-Aldrich) in an LSE™ Benchtop Shaking Incubator (Corning, Corning, NY) at 250 rpm. The next day, bacteria were diluted 1:100 in TSB and grown at 37°C to an optical density at 600 nm (OD_600_) of 0.2 for a bacteria concentration of 1 x 10^8^ colony-forming units (CFU)/mL. For *in vitro* studies, bacteria were pelleted by centrifugation at 5,000 x *g* for 8 minutes at 4°C and subsequently resuspended in sterile PBS at desired concentration for downstream experiments. For *in vivo* studies, bacterial slurries at an OD_600_ of 0.2 were diluted in sterile PBS for a final concentration of 10^4^ CFU/mL, of which 100 μL was administered intratracheally to mice for an estimated inoculum of 10^3^ per mouse. Final bacterial concentrations of the inoculums were confirmed by plating serial dilutions of bacterial slurry on tryptic soy agar (TSA, Sigma-Aldrich) plates and subsequent counting of CFU. For examination of gross morphology, overnight cultures of *Kp* isolates were streaked on brain heart infusion agar plates (Sigma-Aldrich) with 5% sucrose (Sigma-Aldrich) and 0.08% Congo red (Sigma-Aldrich) as previously described by others, and images of bacterial colonies were captured by digital photography after stationary incubation at room temperature for 48 hours (Van Tyne et al., 2016). For *in vitro* growth assays, 1:100 dilutions of overnight bacterial cultures were grown in TSB for 4 hours at 37°C and automated OD_600_ measurements were performed every 20 minutes using a SpectraMax i3x Multi-Mode Microplate Reader (Molecular Devices, San Jose, CA). In parallel, samples of the cultures were taken 0, 2, and 4 hours after starting the 1:100 bacterial cultures. Serial dilutions of these samples were then prepared and plated on TSA plates for CFU quantification.

### pHrodo phagocytosis assay

For pHrodo phagocytosis assays, *Kp* isolates were first heat-killed by incubation of bacteria at 60°C for 1 hour. Heat-killed *Kp* isolates were labeled with pHrodo iFL Green STP ester amine reactive dye (Invitrogen™, Thermo Fisher Scientific) according to the manufacturer’s instructions. Cell culture plates with macrophages were placed on ice for 5 minutes, after which pHrodo-labeled bacteria were added at a multiplicity of infection (MOI) of 10:1 (bacteria:macrophages). Plates were then centrifuged at 400 x *g* for 10 minutes at 4°C, and subsequently, macrophages and bacteria were incubated at 37°C for 1.5 hours to allow phagocytosis. After this, macrophages were collected in ice-cold PBS and the phagocytosis of *Kp* was analyzed directly on a FACSCalibur flow cytometer (BD Biosciences, San Jose, CA). Uptake of *Kp* was assessed based on pHrodo fluorescence and using uninfected cells as a reference to define pHrodo^+^ cells. Macrophages in negative control wells were incubated with cytochalasin D (10 μM) for 1 hour before the addition of *Kp* bacteria. The percentage of pHrodo^+^ cells across macrophages incubated with each of the clinical *Kp* isolates was normalized to fold change compared to macrophages incubated with the reference research strain ATCC 43816 (ATCC).

### Gentamicin protection assay

For assessing phagocytic uptake by gentamicin protection assay, macrophages were incubated with live *Kp* isolates at an MOI of 10:1 in DMEM with 10% heat-inactivated newborn calf serum without antibiotics. After 1 hour, the cell culture medium was aspirated and replaced with DMEM with 10% heat-inactivated newborn calf serum containing 100 μg/mL gentamicin (APP Pharmaceuticals, Schaumburg, IL) for 1 hour to kill remaining extracellular bacteria. Subsequently, macrophages were washed three times with PBS and cells were lysed by incubation with 0.3% Triton X-100 (Sigma-Aldrich) for 15 minutes. To quantify phagocytosis, 10-fold serial dilutions of the macrophage lysates were plated on TSA plates, followed by manual counting of CFUs.

### Scanning electron microscopy

To obtain scanning electron microscopy images, bacteria were grown overnight on agar plates. 12 mm round coverslips were treated with Cell-Tak (Corning). After allowing the solution to dry, the treated side of the coverslip was placed on top of a bacterial colony for 10 minutes. Subsequently, the coverslips were placed into individual wells of 24-well cell culture plates and the bacteria were fixed in 2.5% glutaraldehyde in PBS for 1 hour. Next, the bacteria were washed 3 x 10 minutes in PBS and then post-fixed for 1 hour in 1% aqueous OsO_4_. After this, the bacteria were again washed 3 x 10 minutes in PBS and then dehydrated in 30-100% ethanol series, followed by 2 x 15 minutes in hexamethyldisilazane and air drying. Finally, the coverslips were sputter-coated with 5 nm of gold/palladium alloy (Cressington, Watford, United Kingdom) and imaged on JSM-6330F scanning electron microscope (JEOL, Peabody, MA) at 3 kV.

### Transmission electron microscopy

To obtain scanning electron microscopy images, bacterial colonies grown on agar plates and stained per protocol (Birkhead et al., 2017). Briefly, bacterial colonies were fixed with 2% paraformaldehyde and 2.5% glutaraldehyde in process buffer (0.1 M Na cacodylate, 0.09 M sucrose, 0.01 M CaCl_2_·2H_2_O, 0.01 M MgCl_2_·6H_2_O) supplemented with 1.55% L-lysine acetate (Thermo Fisher Scientific) and 0.075% Ruthenium Red (Thermo Fisher Scientific) for 20 minutes on ice. After this, the bacterial colonies were incubated in fixative in process buffer with ruthenium red (without L-Lysine acetate) for 3 hours at 4°C. The colonies were then washed on ice 3 x 30 minutes with process buffer with ruthenium red (without fixative). After this, the colonies were post-fixed in 1% OsO_4_ in process buffer with ruthenium red for 1 hour on ice and washed 5 x 10 minutes in process buffer with ruthenium red on ice. Colonies were then removed from the agar plate and transferred to glass vials for resin embedding.

For resin embedding, the colonies were washed 3 times in PBS, then dehydrated in 30-100% ethanol series, followed by 100% propylene oxide, and subsequently infiltrated in a 1:1 mixture of propylene oxide:Poly/Bed 812 epoxy resin (Polysciences, Warrington, PA) for 1 hour. After several changes of 100% resin over 24 hours, colonies were embedded in molds and cured at 37°C overnight, followed by hardening at 65°C for 2 more days. Semithin (300 nm) sections of the colonies were heated onto glass slides and subsequently stained with 1% toluidine blue and imaged using light microscopy. Ultrathin (60 nm) cross-sections of the colonies were collected on copper grids and stained with 1% uranyl acetate for 10 minutes, followed by 1% lead citrate for 7 minutes. Sections were imaged using a JEM-1400Flash transmission electron microscope (JEOL) at 80 kV and a bottom mount digital camera (Advanced Microscopy Techniques, Danvers, MA)

### Experimental bacterial pneumonia model

For *in vivo* pulmonary infection studies, mice were inoculated intratracheally with 10^3^ CFU of the clinical *Kp* isolates W42 or S17 through direct visualization as previously described (Zhao et al., 2015). For alveolar macrophage depletion, 100 μL clodronate-containing liposomes (5mg/mL, Encapsula NanoSciences, Brentwood, TN) or empty control liposomes were administered intratracheally 24 hours before infection with *Kp*. Mice were euthanized 24 or 48 hours after infection, at which point bronchoalveolar lavage fluid (BAL) samples were collected by flushing the right lung with PBS containing 0.6 mM EDTA (UltraPure™, Thermo Fisher Scientific) as described in detail previously (Lee et al., 2005). For determination of lung bacterial burden, the left lung of each mouse was collected in 1 mL sterile water and homogenized immediately with a tissue homogenizer. Subsequently, 10-fold serial dilutions of the lung tissue homogenates were plated on TSA plates and incubated overnight at 37°C. The next day, CFUs were determined by manual counting of bacterial colonies.

### BAL and lung tissue measurements

Cytospins for obtaining BAL polymorphonuclear cell counts were prepared from 100-200 μL (~5 x 10^4^ cells) of BAL specimen using a Shandon Cytospin 3 (Thermo Fisher Scientific) at 300 rpm for 3 minutes. Resulting cytospins were stained with Differential Stain (Newcomer Supply, Middleton, WI) according to manufacturer’s instruction. Cell counts and differentials were determined by manually counting 200 consecutive cells as described previously (Lee et al., 2005). Myeloperoxidase activity in lung tissue homogenates was measured as described previously (Qu et al., 2018; Peñaloza et al., 2021) Briefly, hexadecyltrimethylammonium bromide (HTAB, Sigma-Aldrich) in MPO buffer (50 mM KH_2_PO_4_ (Sigma-Aldrich) and 50 mM K_2_HPO_4_ (Sigma-Aldrich) in H_2_O) was added to lung tissue homogenates for a final HTAB concentration of 0.5%, and subsequently, samples were sonicated and centrifuged at 12,000 x *g* for 15 minutes at 4°C. After this, 7 μL of supernatant from each sample was transferred to a 96-well plate and 200 μL assay buffer containing 0.5 mM *O*-dianisidine dihydrochloride (Sigma-Aldrich) and 5% hydrogen peroxide (Sigma-Aldrich) was added to each well. After 1 and 10 minutes, the OD_450_ for each well was measured using a SpectraMax i3x Multi-Mode Microplate Reader (Molecular Devices). MPO activity in each well was calculated by subtracting the absorbance after 1 minute from the absorbance at 10 minutes and dividing the result by 1.13 x 10^-2^. For cytokine measurements, 10X cytokine lysis buffer was added to lung homogenates for final concentrations (1X) of 0.5% Triton X-100, 150 mM NaCl (Sigma-Aldrich), 15 mM Tris (Sigma-Aldrich), 1 mM CaCl (Sigma-Aldrich), 1 mM MgCl (Sigma-Aldrich), pH 7.4, which were then incubated on ice for 30 minutes, and subsequently samples were centrifuged at 10,000 x *g* for 20 minutes at 4°C. Supernatants were then collected and stored at -80°C until analysis of cytokine levels by enzyme-linked immunosorbent assay (ELISA). The levels of TNF, IL-1β, IL-12p40, IL-17A, CXCL1, and CCL5 in lung tissue homogenates were measured using DuoSet ELISA kits (R&D Systems) according to manufacturer’s instructions.

### Flow cytometry analysis of BAL

For assessment of BAL immune cell populations, flow cytometry analysis of BAL samples was performed as described previously (Peñaloza et al., 2021). After obtaining BAL specimens, BAL cells were pelleted by centrifugation at 350 x *g* for 10 minutes at 4°C. BAL cells were then resuspended in ammonium-chloride-potassium (ACK) lysis buffer (150 mM NH_4_Cl (Sigma-Aldrich), 10 mM KHCO_3_ (Sigma-Aldrich), 0.1 mM Na_2_EDTA (Sigma-Aldrich), pH 7.4) and incubated at room temperature for 5 minutes. After this, cells were washed twice with PBS and incubated with LIVE/DEAD™ Fixable Aqua Stain (Invitrogen™, Thermo Fisher Scientific) for 30 minutes at room temperature protected from light. Subsequently, cells were washed twice with PBS and incubated in cell staining buffer (PBS with 2% heat-inactivated newborn calf serum) containing antibodies against extracellular antigens for 30 minutes on ice protected from light. The following anti-mouse antibodies were used for staining: CD45-AF700 (clone: 30-F11, BD Biosciences), CD11b-PE (clone: M1/70, BD Biosciences), Siglec-F-APC-Cy7 (clone: E50-2440, BD Biosciences), CD11c-PE-Cy7 (clone: HL3, BD Biosciences), CD64-BV650 (clone: X54-5/7.1, BD Biosciences), Ly6G-APC (clone: 1A8, BD Biosciences). After staining, cells were fixed by incubation in 2% paraformaldehyde (Sigma-Aldrich) in PBS overnight and stored in PBS at 4°C until time of analysis. CountBright™ Absolute Counting Beads were added to each flow sample to obtain absolute cell counts (Invitrogen™, Thermo Fisher Scientific). Cells were analyzed on an LSRFortessa flow cytometer and data analysis was performed using FlowJo v10 software (BD Biosciences, see **Figure S1** for gating strategy).

### Data analysis

All statistical analyses were performed using Prism 9 software (GraphPad Software, San Diego, CA). Normal probability plots of the residuals were used to evaluate normal distribution of the data. No outliers were excluded from the data. For data that followed a normal distribution, group means are reported, and an unpaired t test with Welch’s correction was applied to test for statistically significant differences between two experimental groups. For data that did not follow a normal distribution, group medians are reported, and a non-parametric Mann-Whitney test was used to detect statistically significant differences. A p-value below 0.05 was considered statistically significant.

## Results

### *Klebsiella pneumoniae* clinical isolates display heterogeneous sensitivity to macrophage phagocytosis *in vitro*

**Figure 1:**
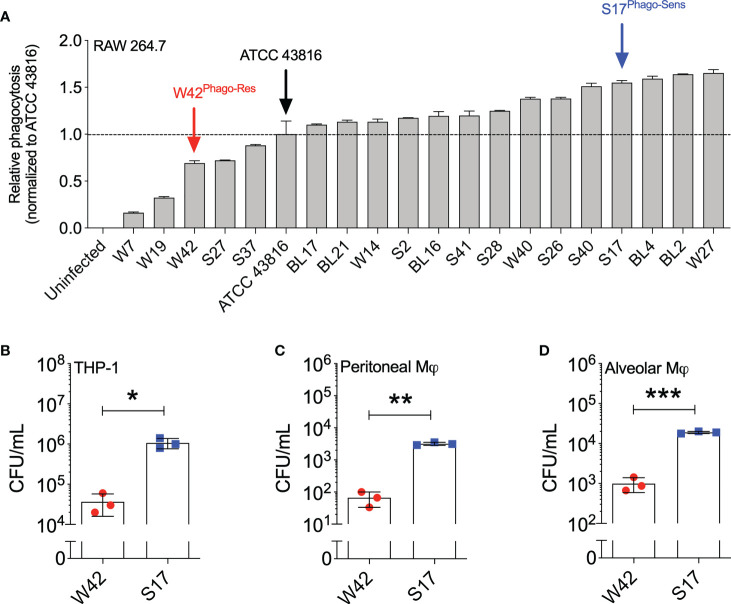
*Kp* clinical isolates show a high variation of phagocytic uptake by macrophages *in vitro*. **(A)** Heat-inactivated clinical *Kp* isolates (N=19) were labeled with pHrodo and incubated with RAW264.7 cells for 1.5 hours (MOI=10; bacteria:macrophage). Subsequently, the phagocytic uptake of *Kp* isolates by macrophages was analyzed by flow cytometry. **(B)** THP-1 cells, **(C)** mouse peritoneal macrophages, and **(D)** mouse alveolar macrophages were incubated with live *Kp* isolates W42 and S17 for 1 hour (MOI=10), after which phagocytic uptake of the bacteria was assessed by gentamicin protection assay. Phagocytosis was quantified by serial dilution of macrophage lysates on TSA plates, followed by manual counting of CFU. Initial screening of the clinical isolates by pHrodo assay in **(A)** was performed in duplicates. Analysis of phagocytic uptake by gentamicin protection assay in **(B–D)** was performed with three technical replicates. Data represent means ± SD **(A)** or means ± SD + individual data points **(B–D)**. *p<0.05, **p<0.01, ***p<0.001 (t test with Welch’s correction).

To examine the extent to which phagocytic uptake varies among clinical isolates of *Kp*, we evaluated the phagocytic uptake of 19 clinical respiratory *Kp* isolates that were previously isolated from hospitalized patients diagnosed with pneumonia (Ito et al., 2015). For this purpose, the *Kp* isolates were heat-killed and labeled with pHrodo, after which the phagocytic uptake of the isolates by RAW264.7 cells was assessed by flow cytometry and compared to the uptake of the reference research strain ATCC 43816 of serotype K2. Using this approach, we observed heterogeneity in phagocytosis sensitivity among the clinical *Kp* isolates, with 5 out of 19 isolates displaying lower phagocytic uptake compared to ATCC 43816, and 14 out of 19 isolates displaying higher phagocytic uptake than ATCC 43816 (**Figure 1A**; **Figure S2**). To verify the observed differences in phagocytic uptake among the clinical *Kp* isolates using a secondary approach, we selected three isolates from the bottom quartile (phagocytosis-resistant; W7, W42, and S27) and two isolates from the top quartile (phagocytosis-sensitive; S17 and W27) and examined their phagocytic uptake by gentamicin protection assay. In this experiment, isolate W42 displayed the highest resistance to macrophage phagocytosis, whereas isolate S17 displayed the highest sensitivity to macrophage phagocytosis. (**Figure S3**). The phagocytic uptake of isolate S17 was also confirmed by gentamicin protection assay to be markedly higher than that of isolate W42 in THP-1 cells, in primary mouse peritoneal macrophages, and in mouse alveolar macrophages, thus demonstrating that the relative phagocytic uptake of W42 (low) and S17 (high) is consistent across macrophage cell lines and primary macrophages (**Figures 1B–D**). Based on these observations, the relatively phagocytosis-resistant W42 isolate (W42^Phago-Res^) and the phagocytosis-sensitive S17 isolate (S17^Phago-Sens^) were selected for further phenotypic characterization of these two clinical isolates with regard to *in vivo* pathogenicity and the host inflammatory response.

### Infection with the phagocytosis-sensitive isolate S17 results in lower lung bacterial burden and reduced inflammation *in vivo* compared to infection with the phagocytosis-resistant isolate W42

**Figure 2:**
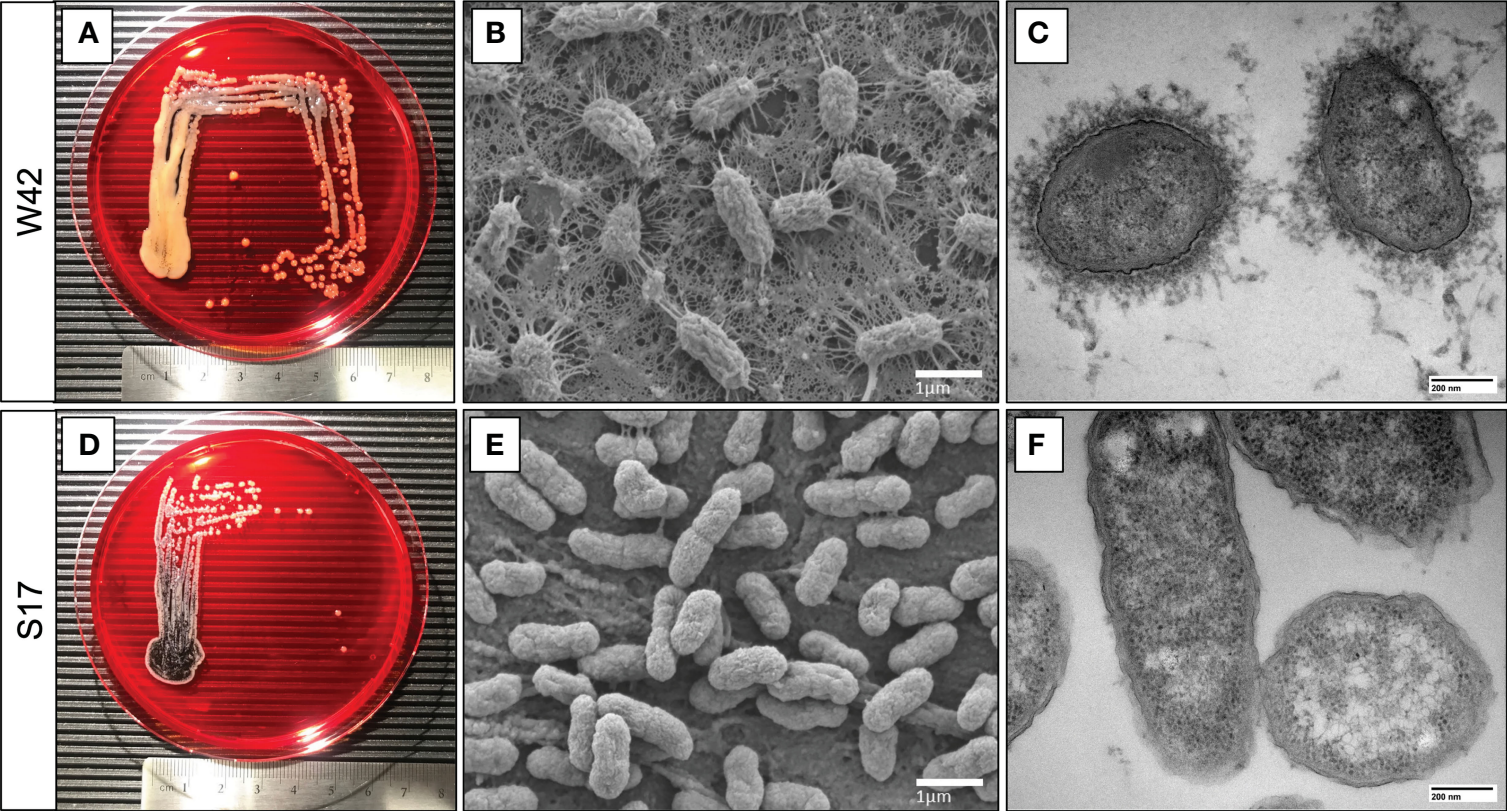
S17 *Kp* isolate displays a non-mucoviscous phenotype compared to the mucoviscous W42 *Kp* isolate. **(A, D)** Representative images of clinical *Kp* isolates W42^Phago-Res^ and S17^Phago-Sens^ grown on congo red agar plates at 37°C for 24 hours, with a centimeter-ruler at the bottom of each image for scale. **(B, E)** Representative scanning electron microscopy images of W42^Phago-Res^ and S17^Phago-Sens^. Scalebar = 1 μm. **(C, F)** Representative transmission electron microscopy images of W42^Phago-Res^ and S17^Phago-Sens^ stained with lanthanum nitrate. Scalebar = 200 nm.

**Figure 3:**
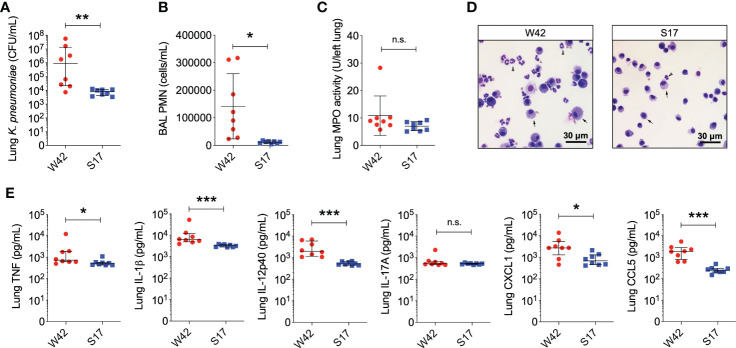
Mice infected with the phagocytosis-sensitive *Kp* isolate S17 display lower bacterial burden accompanied by reduced inflammation compared to mice infected with the phagocytosis-resistant isolate W42. **(A)** CFU of *Kp* in lung homogenates obtained from C57BL/6J mice 48 hours following intratracheal infection with W42^Phago-Res^ (N=8) or S17^Phago-Sens^ (N=8, inoculum: 10^3^ CFU). **(B)** PMN numbers in BAL specimens from mice infected with W42 and S17. **(C)** MPO activity in lung tissue homogenates from W42- and S17-infected mice. **(D)** Representative images of cytospin preparations of BAL specimens from W42- and S17-infected mice stained with a differential stain. Arrow-heads represent PMN, arrows represent mononuclear phagocytes. **(E)** Levels of TNF, IL-1β, IL-12p40, IL-17A, CXCL1, and CCL5 in lung tissue homogenates from W42- and S17-infected mice as determined by ELISA. Data represent medians ± interquartile range **(A, E)** or means ± SD **(B, C)** + individual data points. *p<0.05, **p<0.01, ***p<0.001, n.s.: not significant **(A, E)**: Mann-Whitney test; **(B, C)**: t test with Welch’s correction).

The W42^Phago-Res^ and S17^Phago-Sens^ isolates have been reported previously to be of the K1 serotype and the K57 serotype, respectively (Ito et al., 2015). Macroscopic and electron microscopic examination of W42^Phago-Res^ and S17^Phago-Sens^ showed that W42^Phago-Res^ displays a mucoviscous phenotype with a clearly discernible capsule (**Figures 2A–C**), whereas S17^Phago-Sens^ is a non-mucoid isolate with relatively low capsule production (**Figures 2D–F**). Having identified W42^Phago-Res^ and S17^Phago-Sens^ as isolates with distinct phagocytosis phenotypes and substantial differences in mucoviscosity and capsule production, we next sought to determine how the observed differences in the sensitivity of these two isolates to phagocytic uptake affected the infectivity of these two isolates in a murine model of acute lower respiratory infection. Infection of mice with S17^Phago-Sens^ resulted in a significantly lower bacterial burden 48 hours post-infection compared to infection with a similar inoculum of W42^Phago-Res^ (**Figure 3A**). MPO activity did not significantly differ between lung tissue of mice infected with S17^Phago-Sens^ and mice infected with W42^Phago-Res^, but in line with a lower bacterial burden, mice infected with S17^Phago-Sens^ displayed lower polymorphonuclear leukocyte (PMN) counts in the BAL compared to that in mice infected with W42^Phago-Res^ (**Figures 3B–D**). Furthermore, S17^Phago-Sens^-infected mice showed significantly lower levels of TNF, IL-1β, IL-12p40, CXCL1, and CCL5 in lung tissue compared to W42^Phago-Res^-infected mice, while levels of IL-17A were similar between S17^Phago-Sens^- and W42^Phago-Res^-infected mice (**Figure 3E**). Of note, the W42^Phago-Res^ and S17^Phago-Sens^ isolates displayed similar growth rates *in vitro*, indicating that the lower bacterial burden observed in mice infected with S17^Phago-Sens^ compared to that in mice infected with W42^Phago-Res^ is not due to differences in growth ability between the two isolates (**Figure S4**). Collectively, these findings show that the S17^Phago-Sens^ isolate is more readily cleared by the host than the W42^Phago-Res^ isolate and that this is associated with a lower inflammatory response.

### Alveolar macrophages enhance early host defense against the phagocytosis-sensitive isolate S17 but not the phagocytosis-resistant isolate W42

Alveolar macrophages are considered the sentinel phagocytes in the lung that encounter *Kp* and regulate the innate immune response to the pathogen (Broug-Holub et al., 1997). However, whereas depletion of alveolar macrophages has been shown to impair host defense against the laboratory *Kp* strain ATCC 43816, depletion of alveolar macrophages has been reported to have no effect on the pulmonary clearance of a clinical carbapenem-resistant ST258 *Kp* isolate (Broug-Holub et al., 1997; Ahn et al., 2016). As these reports suggest that the impact of alveolar macrophages on host defense against pulmonary *Kp* infection varies among different strains, we next determined the effect of alveolar macrophage depletion on pulmonary host defense against W42^Phago-Res^ andS17^Phago-Sens^.

**Figure 4:**
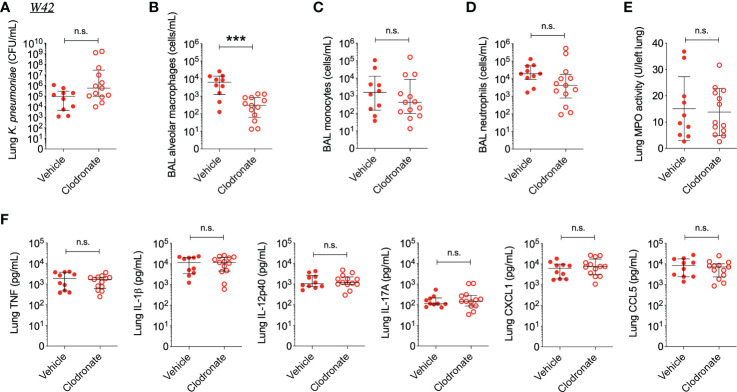
Alveolar macrophage depletion with clodronate does not affect bacterial burden and inflammation in mice infected with the phagocytosis-resistant W42 isolate. **(A)** CFU of *Kp* in lung homogenates obtained from C57BL/6J mice treated with clodronate (N=13, intratracheal, 0.5mg/mouse) or empty liposomes (N=10) and subsequently infected with W42^Phago-Res^ (inoculum: 10^3^ CFU) for 24 hours. **(B)** Numbers of alveolar macrophages, **(C)** monocytes, and **(D)** neutrophils in BAL specimens from W42-infected mice treated with clodronate or vehicle, as determined by flow cytometry. **(E)** MPO activity in lung tissue homogenates from W42-infected mice treated with clodronate or vehicle. **(F)** Levels of TNF, IL-1β, IL-12p40, IL-17A CXCL1, and CCL5 in lung tissue homogenates from W42-infected mice as determined by ELISA. Data represent medians ± interquartile range **(A–D, F)** or means ± SD **(E)** + individual data points. ***p<0.001, n.s.: not significant **(A–D, F)**: Mann-Whitney test; **(E)**: t test with Welch’s correction).

**Figure 5:**
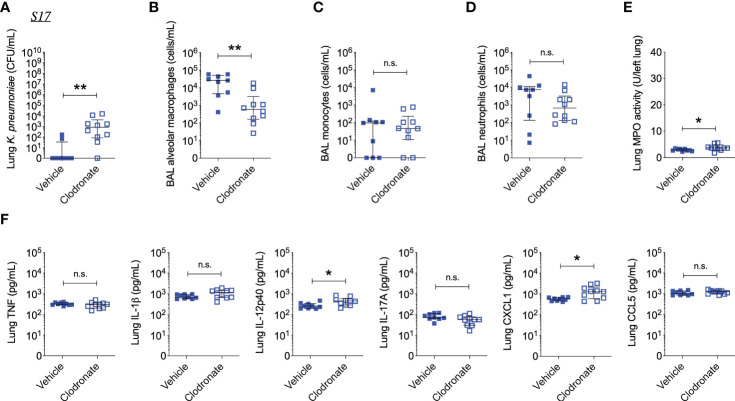
Alveolar macrophage depletion with clodronate results in increased bacterial burden and inflammation in mice infected with the phagocytosis-sensitive S17 isolate. **(A)** CFU of *Kp* in lung homogenates obtained from C57BL/6J mice treated with clodronate (N=10, intratracheal, 0.5mg/mouse) or empty liposomes (N=9) and subsequently infected with S17^Phago-Sens^ (inoculum: 10^3^ CFU) for 24 hours. **(B)** Numbers of alveolar macrophages, **(C)** monocytes, and **(D)** neutrophils in BAL specimens from S17-infected mice treated with clodronate or vehicle, as determined by flow cytometry. **(E)** MPO activity in lung tissue homogenates from S17-infected mice treated with clodronate or vehicle. **(F)** Levels of TNF, IL-1β, IL-12p40, IL-17A, CXCL1, and CCL5 in lung tissue homogenates from S17-infected mice as determined by ELISA. Data represent medians ± interquartile range **(A–D, F)** or means ± SD **(E)** + individual data points. *p<0.05, **p<0.01, n.s.: not significant **(A–D, F)**: Mann-Whitney test; **(E)**: t test with Welch’s correction).

**Figure 6:**
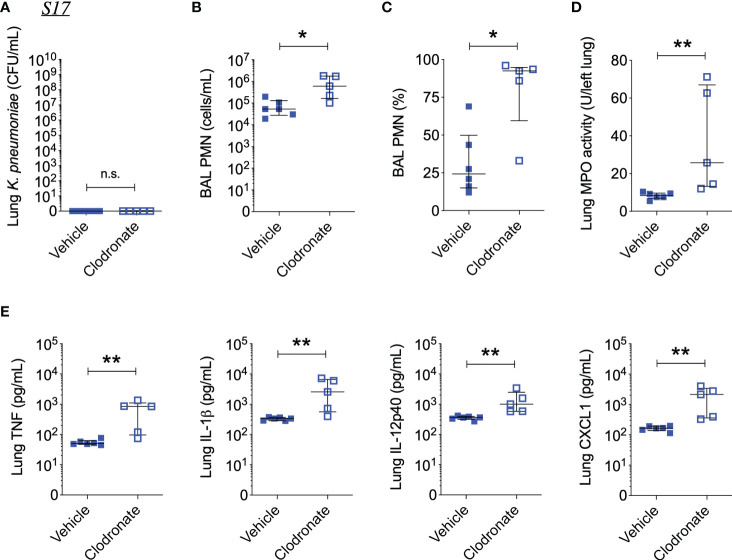
Alveolar macrophage depletion with clodronate is associated with exacerbated inflammation 48 hours after intrapulmonary infection with the phagocytosis-sensitive S17 isolate. **(A)** CFU of *Kp* in lung homogenates obtained from C57BL/6J mice treated with clodronate (N=5, intratracheal, 0.5mg/mouse) or empty liposomes (N=6) and subsequently infected with S17^Phago-Sens^ (inoculum: 10^3^ CFU) for 48 hours. **(B)** Number and **(C)** percentage of PMNs in BAL specimens from S17-infected mice treated with clodronate or vehicle. **(D)** MPO activity in lung tissue homogenates from S17-infected mice treated with clodronate or vehicle. **(E)** Levels of TNF, IL-1β, IL-12p40, and CXCL1 in lung tissue homogenates from S17-infected mice as determined by ELISA. Data represent medians ± interquartile range + individual data points. *p<0.05, **p<0.01, n.s.: not significant (Mann-Whitney test).

Interestingly, clodronate-treatment did not have a significant impact on lung bacterial burden 24 hours following intratracheal infection with W42^Phago-Res^, despite effective depletion of alveolar macrophages from the lungs upon clodronate treatment, as confirmed by flow cytometry analysis of the BAL (**Figures 4A, B**). In line with a similar lung bacterial burden, clodronate-treatment did not appear to affect the host inflammatory response following infection with W42^Phago-Res^, as no differences were observed in the number of monocytes and neutrophils in the BAL, lung MPO activity, and the levels of TNF, IL-1β, IL-12p40, IL-17A, CXCL1, and CCL5 in the lungs of vehicle- and clodronate-treated mice infected with W42^Phago-Res^ (**Figures 4C–F**). In contrast to the observations with W42^Phago-Res^ infection, clodronate-treatment caused a marked increase in bacterial burden 24 hours following intratracheal infection of mice with S17^Phago-Sens^ (**Figures 5A, B**; log10 fold change > 2; p<0.01). Coinciding with this increased bacterial burden, clodronate-treated mice infected with S17^Phago-Sens^ displayed a mild increase in lung MPO activity and lung IL-12p40 and CXCL1 levels at 24 hours post-infection compared to vehicle-treated mice infected with S17^Phago-Sens^, while no differences were observed in BAL monocyte and neutrophil numbers and the levels of TNF, IL-1β, IL-17A, and CCL5 in the lungs of vehicle- and clodronate-treated at the 24 hour timepoint (**Figures 5C–F**). Although clodronate treatment was associated with an increased bacterial burden at the 24-hour timepoint, both vehicle- and clodronate-treated mice were able to clear S17^Phago-Sens^ from the lungs by 48 hours (**Figure 6A**). Despite bacterial clearance by 48 hours, clodronate-treated mice infected with S17^Phago-Sens^ displayed significantly higher neutrophil numbers in the BAL, increased lung MPO activity, and increased levels of TNF, IL-1β, IL-12p40 and CXCL1 in the lungs compared to that in vehicle-treated mice infected with S17^Phago-Sens^ (**Figures 6B–E**). Altogether, these data show that while depleting alveolar macrophages does not significantly affect pulmonary host defense against the phagocytosis-resistant W42 isolate, alveolar macrophages markedly enhance early host defense and restrain the inflammatory response against the phagocytosis-sensitive S17 isolate, thus demonstrating that phagocytic uptake is a critical determinant of the pathogenicity of clinical *Kp* isolates.

## Discussion

In this study, we assessed the *in vitro* phagocytosis sensitivity of 19 clinical respiratory *Kp* isolates and examined phagocytosis susceptibility as a functional correlate of *in vivo Kp* pathogenicity using a phagocytosis-resistant isolate (W42) and a phagocytosis-sensitive isolate (S17). We demonstrate that there is heterogeneity in phagocytosis sensitivity across clinical *Kp* isolates, with 74% of the isolates studied displaying higher phagocytosis-sensitivity than that of the reference *Kp* strain ATCC 43816, and 26% of the *Kp* isolates displaying lower phagocytosis-sensitivity than that of ATCC 43816. *In vivo*, we show that the phagocytosis-sensitive isolate S17 has a markedly lower pathogenicity than the phagocytosis-resistant isolate W42. Moreover, alveolar macrophage depletion reduced pulmonary host defense against the phagocytosis-sensitive isolate S17, while it did not noticeably affect host defense against the phagocytosis-resistant isolate W42. These findings demonstrate that evasion of phagocytosis is a primary determinant of *in vivo Kp* pathogenicity.

Based on our *in vitro* experiments, we identified the W42 isolate as relatively resistant to macrophages phagocytosis compared to the laboratory reference *Kp* strain ATCC 43816, and we report that the depletion of alveolar macrophages does not impact the bacterial clearance of the W42 isolate *in vivo*. This observation is in contrast with our findings with the S17 isolate as well as with a previous report showing that depletion of alveolar macrophages impairs host defense against the laboratory reference *Kp* strain ATCC 43816 (Broug-Holub et al., 1997). However, the fact that AM-depletion did not markedly affect host defense against W42 can likely be explained by our *in vitro* data showing that the W42 isolate evades phagocytic uptake by macrophages, thereby negating any impact of AM-depletion on bacterial clearance. The W42 isolate was previously determined to belong to the K1 serotype (Ito et al., 2015), which is generally considered to be associated with hypervirulence, as is the K2 serotype (Fung et al., 2002; Yeh et al., 2007; Paczosa and Mecsas, 2016). Notably, 2 out of 5 clinical isolates from the top quartile isolates with the highest sensitivity to phagocytic uptake are of the K2 serotype (BL4, and BL2). This observation indicates that the K2 serotype does not necessarily correspond to a phagocytosis-resistant phenotype and, consequently, high pathogenicity. This notion is supported by a report showing that K1/K2 isolates and non-K1/K2 isolates that are positive for the hypermucoviscosity phenotype, *rmpA* and aerobactin genes display similar *in vivo* virulence irrespective of their capsular serotype, which emphasizes that caution is warranted when designating *Kp* isolates as hypervirulent based on K1/K2 serotyping alone (Yu et al., 2008). As increased capsule production has previously been shown to facilitate evasion of phagocytosis by *Kp* (Yoshida et al., 2000; Cortés et al., 2002; March et al., 2013), our electron microscopic images showing that W42 exhibits a mucoviscous phenotype provide a possible explanation for the phagocytosis resistance of the W42 isolate, although mechanistic studies with CPS-deficient mutants of W42 would be required to determine that this is indeed the case. In addition, it is important to note that factors other than capsular polysaccharide production could also be responsible for or contributing to the observed differences in phagocytic uptake between the W42 and S17, such as differences in lipopolysaccharide (LPS), outer membrane proteins (OMP), and fimbriae (Athamna and Ofek, 1988; March et al., 2013).

In contrast to our observations with W42, we identified the S17 *Kp* isolate as possessing a non-mucoviscous phenotype and sensitive to macrophages phagocytosis compared to the laboratory reference *Kp* strain ATCC 43816. *In vivo*, the phagocytosis-sensitive S17 isolate was markedly less pathogenic than W42, and alveolar macrophage-depleted mice displayed a higher bacterial burden 24 hours following infection with S17. This finding is in line with a prior report showing that alveolar macrophage-depletion decreases bacterial clearance of ATCC 43816 and supports the notion that phagocytosis is a functional correlate of *in vivo* pathogenicity of clinical *Kp* isolates (Broug-Holub et al., 1997). Interestingly, both control and alveolar macrophage-depleted mice were able to clear S17 from the lungs by 48 hours after the start of the infection, but alveolar macrophage-depleted mice displayed an increased inflammatory response compared to control mice at this timepoint. One possible explanation for the elevated inflammatory status in alveolar macrophage-depleted mice is that this is a direct result of the increased bacterial burden observed in these mice 24 hours post-infection. Another possibility is that alveolar macrophages play an important role in regulating the host inflammatory response following bacterial clearance, and depletion of alveolar macrophages leads to an exacerbated inflammatory response that would otherwise be curtailed by alveolar macrophages. As prior studies have shown that alveolar macrophages are important contributors to the resolution of inflammation in a variety of contexts (Valstar et al., 2006; Bang et al., 2011; Westphalen et al., 2014; Bourdonnay et al., 2015; Speth et al., 2016; Allard et al., 2018), we favor the explanation that the heightened inflammation in the lungs of AM-depleted mice at 48 hours is a direct consequence of the absence of alveolar macrophages that normally curtail the inflammatory response.

Although our study does not provide experimental data that directly addresses what the source of inflammatory cytokine and chemokine production is in absence of alveolar macrophages, available data from other studies indicate that epithelial cells and recruited monocytes are likely involved in this process. Several studies, for example, have shown that airway epithelial cells can produce CXCL1 and CCL5 in response to inflammatory stimuli (Wang et al., 1996; Olszewska-Pazdrak et al., 1998; Casola et al., 2002; Elizur et al., 2007; Cai et al., 2010; Farnand et al., 2011; Chuquimia et al., 2012; Sharma et al., 2014). Importantly, both of these chemokines are known to mediate recruitment of neutrophils and monocytes, which can thus also account for our observation that there is no deficiency in neutrophil and monocyte recruitment in alveolar macrophage-depleted mice (Schall et al., 1990; Pan et al., 2000; Smith et al., 2005; Sawant et al., 2015; Paudel et al., 2019). It is further worth noting that monocytes are a known source of IL-12p40, and that recruited inflammatory monocytes have been shown to be an important source of TNF and – indirectly – IL-17A in the lungs of *Kp*-infected mice (D’Andrea et al., 1992; Brown et al., 2016; Xiong et al., 2016). This provides an explanation for our observation that the production of TNF, IL-12p40, and IL-17A is not impaired in the lungs of alveolar macrophage-depleted mice.

Given the observed heterogeneity in phagocytosis susceptibility among clinical *Kp* isolates, an area of interest for future research is the evaluation of phagocytosis sensitivity among clinical isolates of other respiratory pathogens, such as *Pseudomonas aeruginosa* and *Streptococcus pneumoniae*, which are both also a common cause of hospital-acquired bacterial pneumonia (Magill et al., 2018). With regard to *S. pneumoniae*, capsule-deficient mutants have been shown to be more susceptible to phagocytic uptake compared to wild-type control bacteria, but comparison of phagocytosis sensitivity across a large number of clinical respiratory isolates of *S. pneumoniae*, and examination of how this impacts their initial clearance by macrophages has not been performed (Hyams et al., 2010). Similarly, there is limited data available on whether or not there is heterogeneity in phagocytosis sensitivity among clinical respiratory isolates of *P. aeruginosa*. With regard to phagocytosis of *P. aeruginosa* it is worth noting, however, that persistent *P. aeruginosa* infections in cystic fibrosis patients are strongly associated with the loss of bacterial motility over time, which results in remarkable resistance to phagocytic uptake (Mahenthiralingam et al., 1994; Amiel et al., 2010). This observation raises the question whether *Kp* within patients with recurrent infections can also undergo phenotypic adaptations that affect phagocytosis sensitivity, but to the best of our knowledge this has not yet been investigated experimentally.

In conclusion, we show there is heterogeneity in the sensitivity of clinical *Kp* isolates to phagocytic uptake by macrophages *in vitro*, and that the phagocytosis-sensitive isolate S17 has a lower pathogenicity *in vivo* compared to the relatively phagocytosis-resistant isolate W42. In addition, we show that alveolar macrophages contribute to host defense against the phagocytosis-sensitive S17 isolate, while having minimal impact on the clearance of the phagocytosis-resistant W42 isolate. These data provide experimental support for the notion that phagocytosis is a functional correlate of *in vivo Kp* pathogenicity and that evasion of phagocytosis is an important virulence strategy among clinical respiratory *Kp* isolates. These observations contribute to a better understanding of the host-pathogen interactions that affect immune effectiveness against *Kp* and may ultimately help to improve strategies for combatting complications from increasingly antibiotic-resistant respiratory *Kp* infections.

## Data availability statement

The original contributions presented in the study are included in the article/**Supplementary Material**. Further inquiries can be directed to the corresponding author.

## Ethics statement

The animal study was reviewed and approved by the University of Pittsburgh Institutional Animal Care and Use Committee.

## Author contributions

RG, HF, HP, MH, and JL conceived and designed the research. RG, HF, HP, WB, ZX, NK, EL, MS, JF and DS performed the experiments. RI provided critical reagents. RG, HF, HP, WB, EL, KC, MH and JL analyzed the data. RG, HF, HP, WB, EL, KC, MH and JL interpreted the results of the experiments. RG, and HF prepared the figures. RG, MH, and JL drafted the manuscript. RG, WB, EL, RI, YD, MH, and JL edited and revised the manuscript. All authors contributed to the article and approved the submitted version.
