# Supplementary material for: Phagocytosis is a primary determinant of pulmonary clearance of clinical Klebsiella pneumoniae isolates
Source: Front Cell Infect Microbiol. 2023 Mar 28;13:1150658. doi: 10.3389/fcimb.2023.1150658 (PMC10086180; doi:10.3389/fcimb.2023.1150658)
Supplement: Supplementary file 1 [file DataSheet_1.pdf]

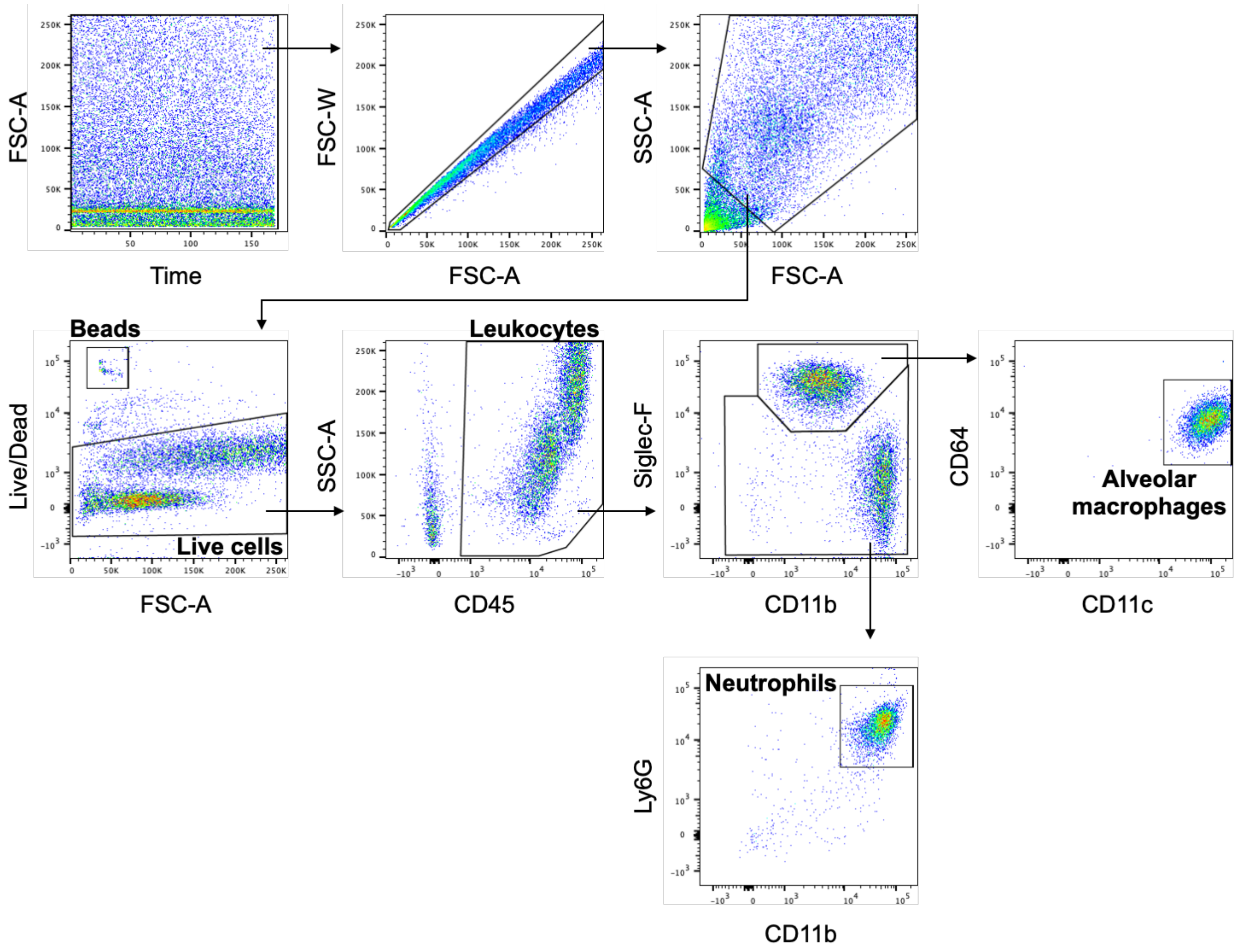

Figure S1. Gating strategy used for the identification of alveolar macrophages and neutrophils in BAL samples by flow cytometry. Live cells were identified using the Live/Dead Fixable Aqua Dead Cell Stain. Cells were stained for extracellular antigens using the following antibodies: CD45-AF700 (clone: 30-F11), CD11b-PE (clone: M1/70), Siglec-F-APC-Cy7 (clone: E50-2440), CD11c-PE-Cy7 (clone: HL3), CD64-BV650 (clone: X54-5/7.1), Ly6G-APC (clone: 1A8), Gating of alveolar macrophages and neutrophils was performed as described previously (32). CountBrightTM Absolute Counting Beads were used to obtain absolute cell counts.
