# Supplementary material for: Phagocytosis is a primary determinant of pulmonary clearance of clinical Klebsiella pneumoniae isolates
Source: Front Cell Infect Microbiol. 2023 Mar 28;13:1150658. doi: 10.3389/fcimb.2023.1150658 (PMC10086180; doi:10.3389/fcimb.2023.1150658)
Supplement: Supplementary file 2 [file DataSheet_2.pdf]

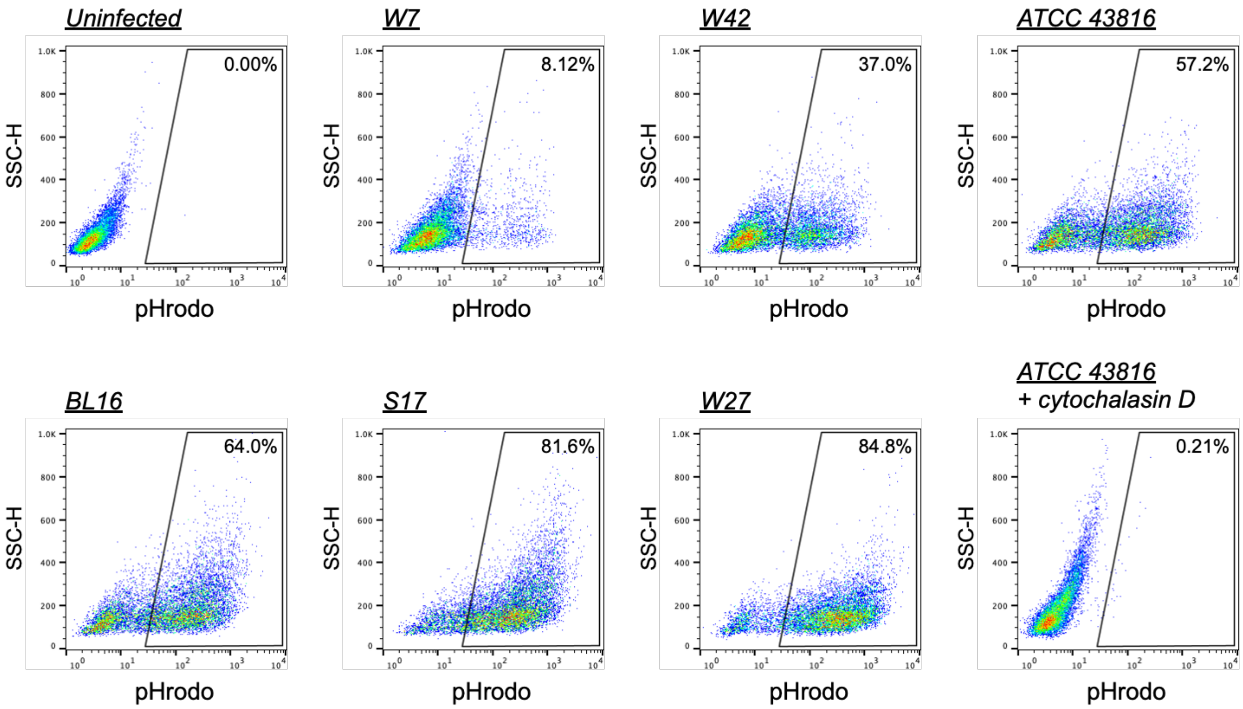

Figure S2. Representative flow plots of macrophages incubated with pHrodo-labeled clinical *Kp* isolates. Heat-inactivated clinical *Kp* isolates (N=19) were labeled with pHrodo and incubated with RAW264.7 cells for 1.5 hours (MOI=10). Subsequently, the phagocytic uptake of *Kp* isolates by macrophages was analyzed by flow cytometry. Shown are representative flow plots of macrophages incubated with *Kp* isolates W7, W42, ATCC 43816 (laboratory reference), BL16, S17, W27, and macrophages pretreated with cytochalasin D (10 μM; negative control) before incubation with ATCC 43816.
