# Supplementary material for: Phagocytosis is a primary determinant of pulmonary clearance of clinical Klebsiella pneumoniae isolates
Source: Front Cell Infect Microbiol. 2023 Mar 28;13:1150658. doi: 10.3389/fcimb.2023.1150658 (PMC10086180; doi:10.3389/fcimb.2023.1150658)
Supplement: Supplementary file 3 [file DataSheet_3.pdf]

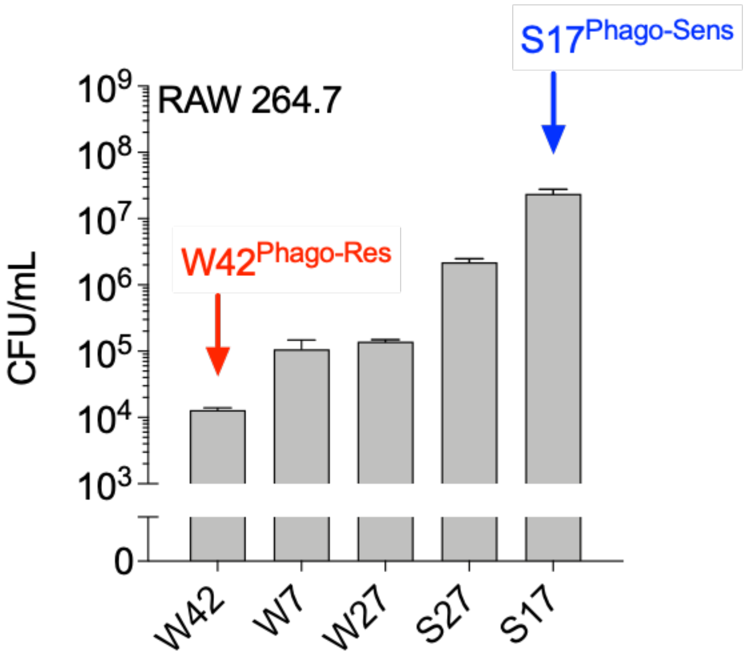

Figure S3. Phagocytosis resistance of W42 and phagocytosis sensitivity of S17 are consistent in a gentamicin-based phagocytosis assay. RAW264.7 were incubated with live *Kp* isolates W7, W42, S27, S17, and W27 for 1 hour (MOI=10), after which phagocytic uptake of the bacteria was assessed by gentamicin protection assay. Phagocytosis was quantified by serial dilution of macrophage lysates on TSA plates, followed by manual counting of CFU. Experiment was performed with three technical replicates for each strain. Data represent means ± SD.
