# Supplementary material for: Phagocytosis is a primary determinant of pulmonary clearance of clinical Klebsiella pneumoniae isolates
Source: Front Cell Infect Microbiol. 2023 Mar 28;13:1150658. doi: 10.3389/fcimb.2023.1150658 (PMC10086180; doi:10.3389/fcimb.2023.1150658)
Supplement: Supplementary file 4 [file DataSheet_4.pdf]

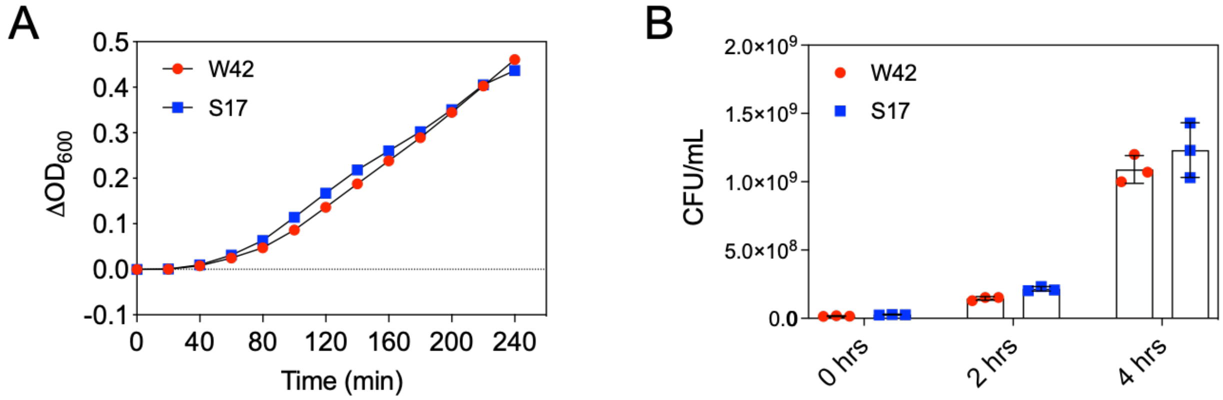

Figure S4. The phagocytosis-resistant W42 isolate and the phagocytosis-sensitive S17 isolate display similar growth rates *in vitro*. (A) Optical density at 600 nm (OD_{600}) measurements of clinical *Kp* isolates $W42^{Phago\text{-}Res}$ and $S17^{Phago\text{-}Sens}$ grown in TSB at 37°C for 4 hours at a 1:100 dilution from overnight bacterial cultures. ΔOD_{600} represents the change in OD_{600} at each timepoint relative to time=0. **(B)** Quantification of colony-forming units (CFU) in samples taken from W42 and S17 cultures at 0, 2, 4 hours of culture in TSB at 37°C. Assay was performed with replicates started from three separate overnight cultures of W42 and S17. Data represent means ± SD (A) or means ± SD + individual data points (B).
